# Adjacent segment infection after surgical treatment of spondylodiscitis

**DOI:** 10.1007/s10195-015-0380-9

**Published:** 2015-10-24

**Authors:** Ahmed Ezzat Siam, Hesham El Saghir, Heinrich Boehm

**Affiliations:** Department of Spinal Surgery, Zentralklinik Bad Berka, Bad Berka, Germany; Spine Unit, El Hadara University Hospital, Alexandria, Egypt; Department of Spinal Surgery with Scoliosis Centre, Schön Klinik Vogtareuth, Krankenhausstrasse 20, 83569 Vogtareuth, Germany

**Keywords:** Adjacent segment infection, Spondylodiscitis, Spondylitis, Spinal infection, Adjacent segment disease

## Abstract

**Background:**

This is the first case series to describe adjacent segment infection (ASI) after surgical treatment of spondylodiscitis (SD).

**Materials and methods:**

Patients with SD, spondylitis who were surgically treated between 1994 and 2012 were included. Out of 1187 cases, 23 (1.94 %) returned to our institution (Zentralklinik Bad Berka) with ASI: 10 males, 13 females, with a mean age of 65.1 years and a mean follow-up of 69 months.

**Results:**

ASI most commonly involved L3–4 (seven patients), T12–L1 (five) and L2–3 (four). The mean interval between operations of primary infection and ASI was 36.9 months. All cases needed surgical intervention, debridement, reconstruction and fusion with longer instrumentation, with culture and sensitivity-based postoperative antimicrobial therapy. At last follow-up, six patients (26.1 %) were mobilized in a wheelchair with a varying degree of paraplegia (three had pre-existing paralysis). Three patients died within 2 months after the ASI operation (13 %). Excellent outcomes were achieved in five patients, and good in eight.

**Conclusions:**

Adjacent segment infection after surgical treatment of spondylodiscitis is a rare complication (1.94 %). It is associated with multimorbidity and shows a high mortality rate and a high neurological affection rate. Possible explanations are: haematomas of repeated micro-fractures around screw loosening, haematogenous spread, direct inoculation or a combination of these factors. ASI may also lead to proximal junctional kyphosis, as found in this series. We suggest early surgical intervention with anterior debridement, reconstruction and fusion with posterior instrumentation, followed by antimicrobial therapy for 12 weeks.

**Level of evidence:**

Level IV retrospective uncontrolled case series.

## Introduction

Spondylodiscitis (SD) is a rare disease with incidence varying globally from one per 100,000 to one per 250,000/year [1, 2]. In many patients, clinical and imaging findings suggest the diagnosis before microbiological confirmation is obtained, and a causative organism remains unknown in up to 40 % of patients [2–4], causing greater difficulty for physicians in selecting the most appropriate antimicrobial treatment [5].

Although an elevation in C-reactive protein (CRP) and/or erythrocytic sedimentation rate (ESR) should not be taken as pathognomonic for an infection, both serve as screening and surveillance tests in the diagnosis and treatment of spinal infections [6]. The high sensitivity, specificity and accuracy of magnetic resonance imaging (MRI) make it the main imaging diagnostic tool in spinal infection [7]. A clinical diagnosis of spondylitis can be made in patients with positive blood cultures and compatible clinical history in combination with corresponding changes on laboratory and imaging studies. A definitive diagnosis of spondylitis can only be made on microscopic or bacteriological examination and culture of infected tissues [8].

Pyogenic infection in the postoperative period is a well documented complication of spinal surgery. In this case, the infection occurs mainly in the operated spinal segment. Adjacent segment infection (ASI) is a very uncommon complication [9].

To the best of the authors’ knowledge, no previously published study has described ASI after surgical treatment of SD. The current study aims to report and discuss this rare phenomenon.

## Materials and methods

### Study design

Single-centre, multi-surgeon, retrospective study of clinical and radiological outcome measures.

### Patients

**Fig. 1:**
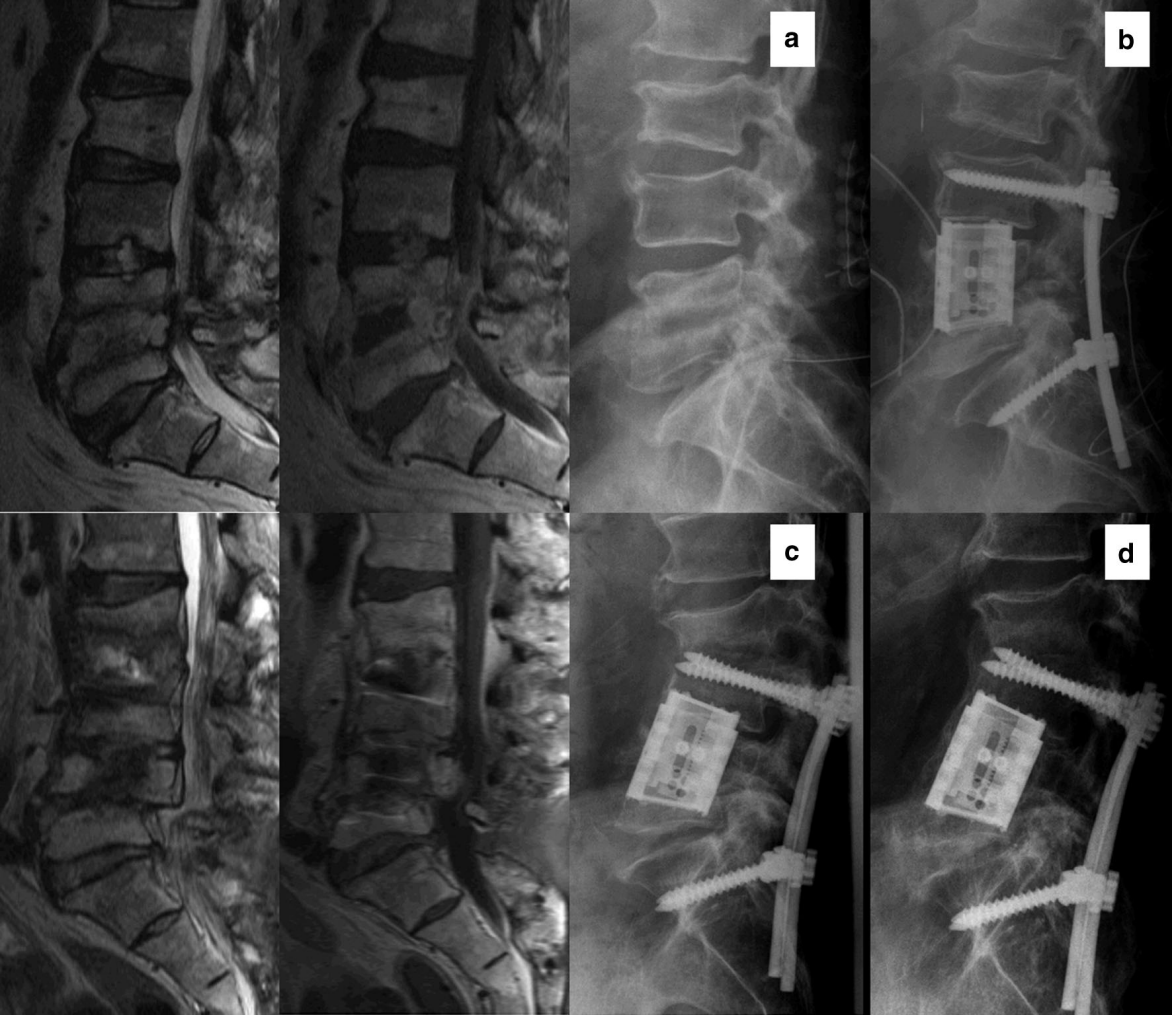
Patient 4: sagittal MRI cuts T2- and T1-weighted, and lateral radiographs; **a** preoperatively, **b** after primary operation, **c** adjacent segment infection, screw loosening and marked adjacent segment kyphosis and **d** last FU after 5.5 years

**Fig. 2:**
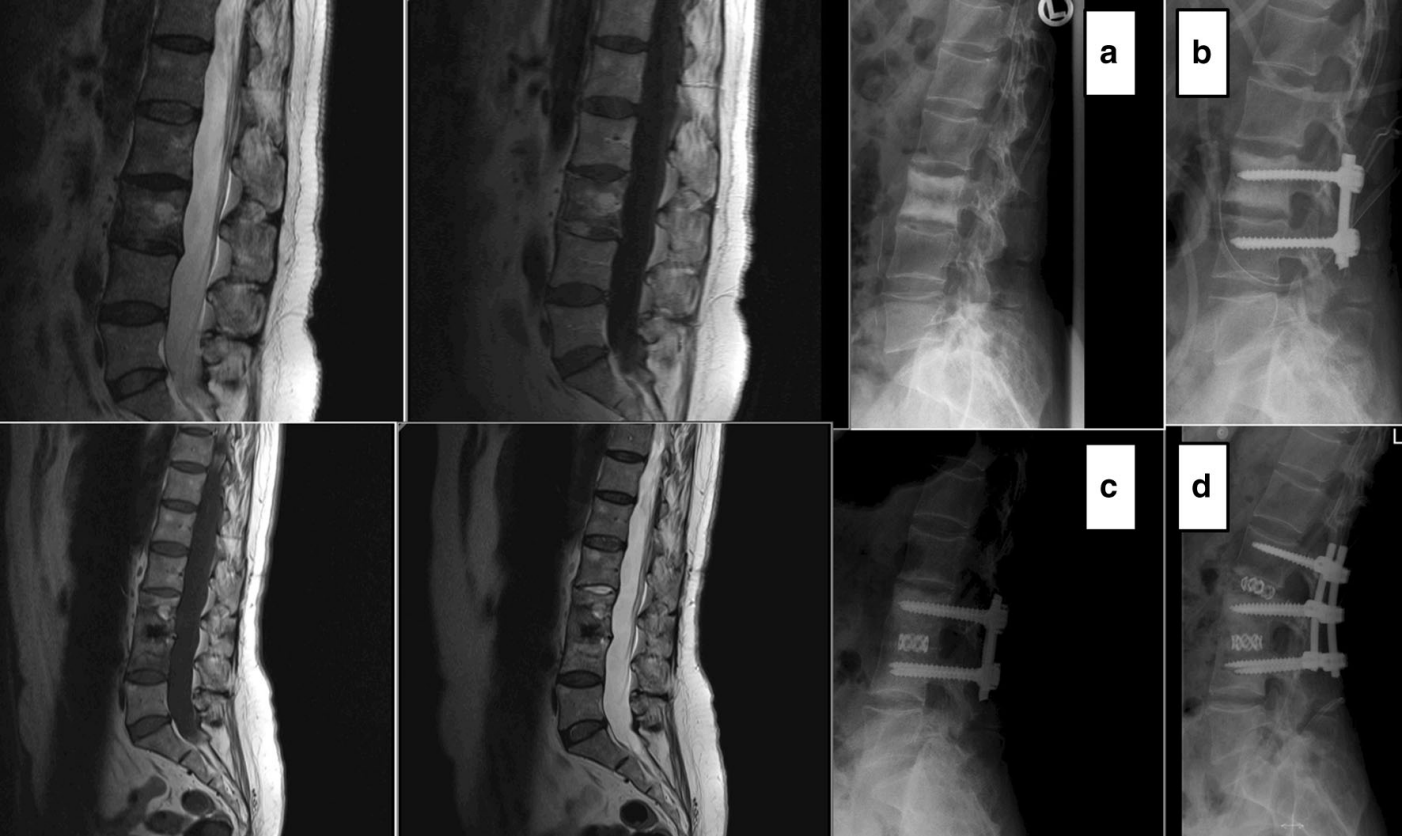
Patient 18: **a** preoperative MRI and radiographs, **b** postoperative, **c** ASI in MRI and radiographs and **d** after reoperation

**Fig. 3:**
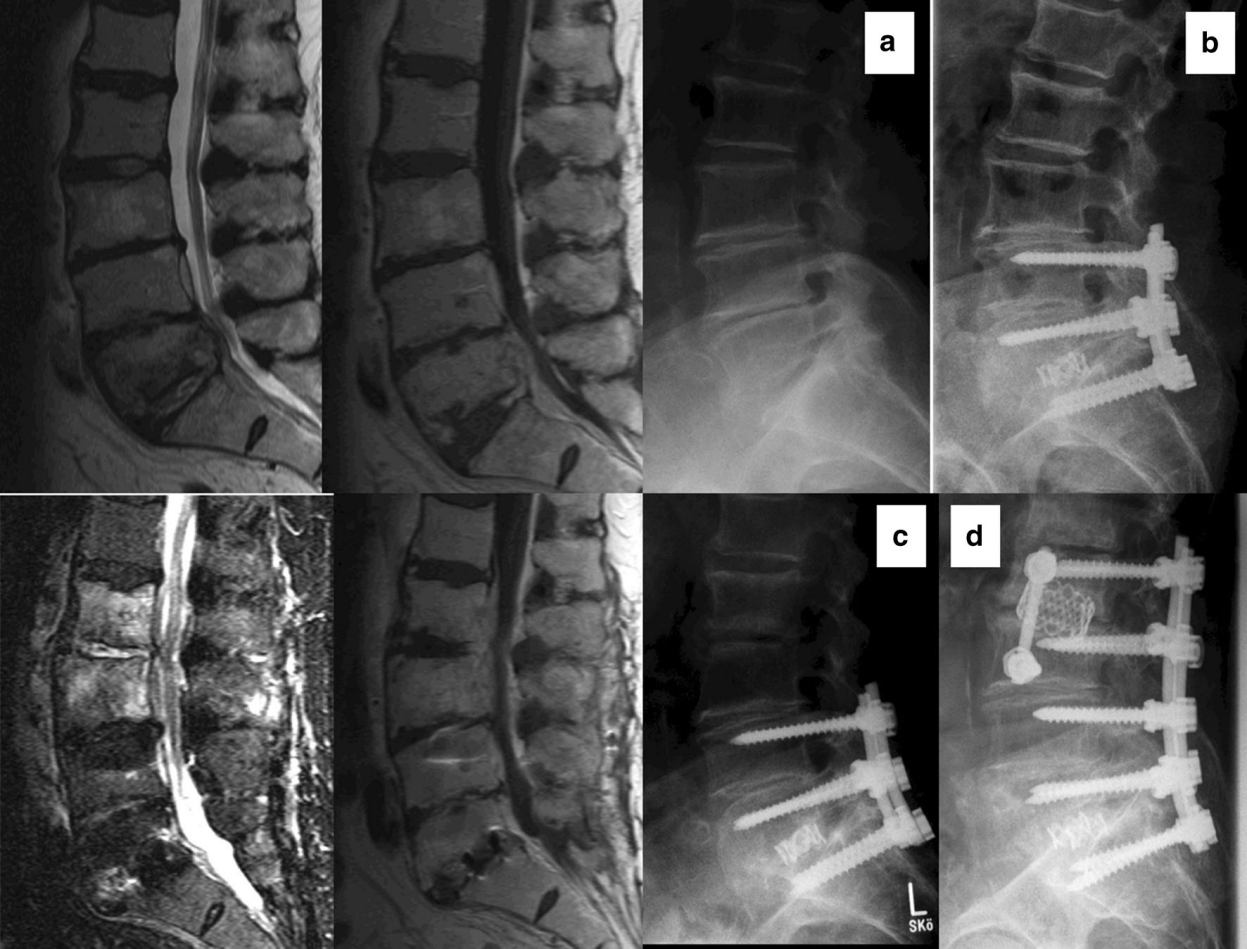
Patient 8: sagittal MRI cuts T2- and T1-weighted, and lateral radiographs; **a** preoperatively, **b** after primary operation, **c** adjacent segment infection, no screw loosening or marked adjacent segment kyphosis, and **d** last FU after 2.5 years

**Fig. 4:**
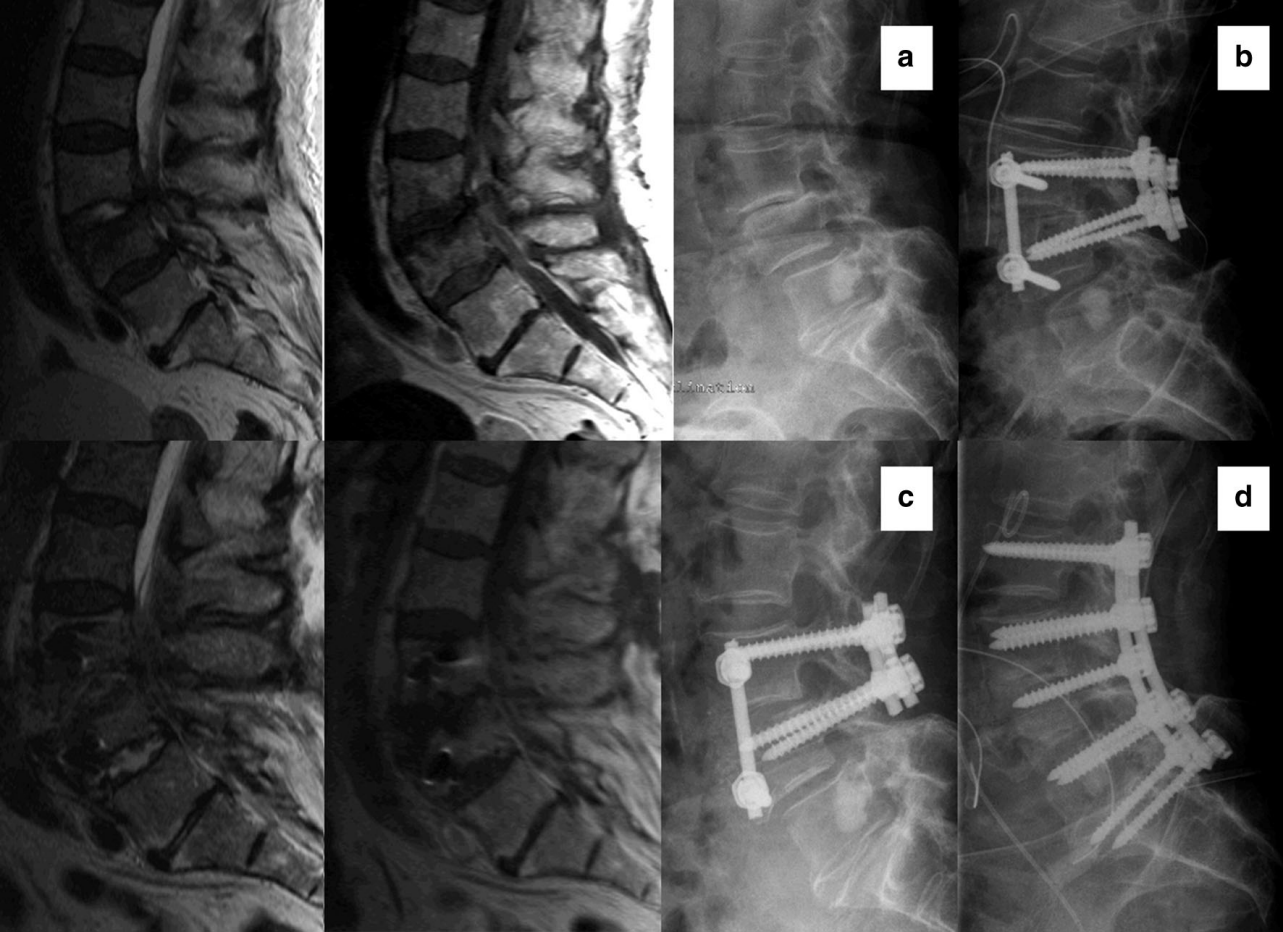
Patient 12: sagittal MRI cuts T2- and T1-weighted, and lateral radiographs; **a** preoperatively, **b** after primary operation, **c** adjacent segment infection, and **d** after ASI surgery

The medical database of our institution (Zentralklinik Bad Berka) was reviewed for patients with spinal infection who were surgically treated from 1994 to 2012. Patients with ASI were included. Patients with same level recurrent infection were excluded, as well as patients with ASI after surgery for spinal pathologies other than SD. Data were collected regarding demographics, presenting signs and symptoms, and predisposing and risk factors (Table 1). We also collected information regarding the level(s) of spinal involvement, perioperative inflammatory markers [white blood cell count (WBC), erythrocyte sedimentation rate (ESR), C-reactive protein (CRP)], microbiological examination (blood cultures, intra-operative biopsy) and imaging modalities. Routinely, plain radiographs in anteroposterior and lateral views, and magnetic resonance imaging (MRI) of the whole spine routinely T1- and T2-weighted with and without contrast medium were performed. Additionally, computed tomography (CT) imaging was done in cases of marked bone destruction. This review also included the management of this phenomenon as regards antimicrobial treatment and surgical intervention, as well as surgical data, complications and outcomes (Figs. 1, 2, 3, 4).

**Table 1:** Demography, comorbidities, primary presentation, neurological function and FU outcomes

Patient	Age (years)/sex	Risk factors, previous operations, co-morbidities and co-infections	ASA score	Main presentation	Duration of symptoms (months)	WBC (/mm^3^)	ESR (mm/h)	CRP (mg/dL)	Frankel grade (primary)	Frankel grade (ASI)	FU Frankel grade	FU after primary operation (months)	Final outcome (Odom’s criteria)
1	69.4/M	MO, DM, BPH, HT, AA, renal stones, hypothyroidism. ASI: fever, UTI, sepsis	4	Fever	1.5	18.4	94	226.7	E	E	E	59.4	Good
2	68.8/F	DM, HT, PNP, heel ulcers, UTI, TKR	3	Weakness	2 weeks	8.5	65	42.2	C	C	C	45.7	Good
3	40.8/F	MO	1	BP	1	9.8	90	94.8	E	E	E	182	Excellent
4	55.1/M	Alcoholism, paraplegia sub-L2	2	Weakness	5	12.3	68	139.1	C	C	C	67.3	Fair
5	50.6/M	DM, HT	3	Paraplegia	1.5	7.8	93	103.1	B	B	B	13.1	Died MOF, septic shock (40 days)
6	70.8/F	HT, breast cancer, OP, IHD	4	BP	5	9.7	84	229.7	E	C	B	16.5	Died MOF, pneumonia, septicemia (16 days)
7	75.6/F	Poliomyelitis, Parkinsonism, MO, HT, DM, IHD, hypothyroidism	4	BP	5	9.1	17	5	C	C	C	32.7	Good
8	77.8/F	Sigmoid colon cancer, depression, hypothyroidism, MO, OP, HT, UTI	4	BP	1.5	4.8	48	45.9	E	E	E	30.3	Died GIT bleeding
9	81.1/M	PLS, re-infection	1	BP	1	10.8	100	172	E	E	E	93.8	Excellent
10	56/F	Alcoholism, alcoholic liver cirrhosis, chronic anaemia	3	BP	1 week	10.3	48	67.4	E	E	E	4.8	Good
11	47.6/M	HT	2	Weakness	1 day	2.6	91	45.9	D	C	C	73.2	Died MOF, sepsis MRSA (29 days)
12	75.9/F	DM, IHD, OP	3	BP	3 weeks	10	110	267	E	E	E	95.8	Excellent
13	69.8/F	Septicaemia, shoulder infection, fever	4	BP	2.5	20.9	135	155.8	E	E	E	38	Excellent
14	57.7/F	Hypothyroidism, hip abscess	2	Paraparesis	2 weeks	10.5	66	7.9	C	E	E	130.5	Excellent
15	73/M	HT	2	BP	3.5	7.2	70	72.2	E	D	C	30	Fair
16	65.7/M	Parkinsonism, UTI	2	BP	1	7	4	5	E	E	C	70.3	Fair
17	73.1/M	Chronic respiratory insufficiency, HT, paraplegia, OP, IHD, hemiparesis, vertebral fracture	4	BP	1	5.8	140	121.8	C	C	B	10.2	Poor
18	55.7/F	Bronchial asthma, re-infection	2	BP	4	8.1	31	11.2	E	E	E	87.5	Good
19	69.9/M	DM, HT, cortison, RI, HI. ASI: septicaemia, fever, toe infection	4	Weakness	6 days	6.5	69	74.8	C	C	D	46.4	Good
20	80.2/F	OP, HT, MVR, UTI, HBV, cholecystectomy	3	BP	1	7.1	104	113	E	D	D	36.2	Good
21	57.7/M	IHD, MO, HT, RI. ASI: septicaemia	3	BP	4	10.9	140	31	E	E	E	58	Good
22	65.1/F	RA, foot infection, cholecystectomy, cortisone. ASI: septicaemia, UTI	3	BP	6	21.7	88	138	E	D	D	210.3	Died MOF (after long intensive care and multiple wound infetions)
23	59.6/F	DM, RI, HT, bilateral TKR, ovarian cancer operation, chemotherapy	3	BP	1 week	6.3	35	10.1	E	E	E	156	Fair

*M* male, *F* female, *ASA* American Society of Anaesthesiology (general condition), *MO* morbid obesity, *DM* diabetes mellitus, *BPH* benign prostatic hyperplesia, *HT* hypertension, *AA* aortic aneurysm, *ASI* adjacent segment infection, *UTI* urinary tract infection, *PNP* polyneuropathy, *TKR* total knee replacement, *OP* osteoporosis, *IHD* ischaemic heart disease, *PLS* postlaminectomy syndrome, *RI* renal insufficiency, *RA* rheumatoid arthritis, *BP* back pain, *WBC* white blood cell count, *ESR* erythrocyte sedimentation rate, *CRP* C-reactive protein, *FU* follow-up, *MOF* multiorgan failure, *MRSA* methicillin-resistant *Staphylococcus aureus*

The general condition of the patient was categorized according to the American Society of Anesthesiologists (ASA) score [10]. The ASA score is a subjective assessment of a patient’s overall health that is based on five classes:

1. Patient is completely healthy and fit.
2. Patient has mild systemic disease.
3. Patient has severe systemic disease that is not incapacitating.
4. Patient has incapacitating disease that is a constant threat to life.
5. A moribund patient who is not expected to live for 24 h with or without surgery.

Data collection, assessment of the radiological findings and statistical analysis were performed by the first author (AES) and critically revised by the others. The adjacent segment lordosis angle was measured between the endplates above and below on the lateral views of postoperative radiographs and compared to those at the time of presentation with ASI (Table 2).

**Table 2:** Patient data at admission and possible explanations of adjacent segment infection

Patient	Interval before ASI surgery (months)	ASI level(s)	Presentation (ASI)	WBC (/mm^3^)	ESR (mm/h)	CRP (mg/dL)	Septicaemia	Screw loosening	Change in ASK
1	5.3	L3–4	Fever	12.7	54	86.2	Yes (*Escherichia coli*)	No	–
2	19.6	L3–4, L5–S1	BP	11.7	132	93.7	No	Yes (+cage)	15°
3	11.9	L3–4	BP	10	83	99	No	No	–
4	14.4	L2–3	Decubitus ulcer	7.5	57	38.2	No	Yes	28°
5	6	T11–12	BP	10.2	73	240.7	Yes (MRSA)	Yes	26°
6	8.6	T12–L1	BP	7.1	94	71.4	Yes (*Pseudomonas aeruginosa* MDR)	Yes	17°
7	16.8	L3–4	BP	5.1	17	3	No	No	–
8	12.3	L2–3	BP	9.5	61	55.4	No	No	–
9	61.1	T12–L1	BP	10.3	84	203	No	Yes	–
10	14 days	L3–4	BP	14.3	85	84.4	No	No	–
11	72.3	L1–L2	Weakness	2.4	81	62.7	Yes (*Staphylococcus aureus*)	Yes	–
12	86.2	L4–5	BP	5.7	97	38.9	No	No	–
13	27.4	T12–L1	BP	7	84	45.5	Yes (*Staphylococcus aureus*)	No	–
14	6	L1–2	BP	14.3	140	180	No	Yes	–
15	17.4	L1–2	Cauda equina syndrome	8.9	90	44.8	No	Yes	–
16	39	T11–L1	BP	6	5	1.9	No	No	–
17	4.5	L3–4	Wound infection	10.2	140	274.4	No	Yes	10°
18	46	L2–3	BP	5.2	37	7.7	No	No	–
19	25.9	T1–2	Fever	23.4	105	270.2	Yes (*Staphylococcus aureus*)	No	–
20	9.9	L2–3	BP	34	47	63.5	No	Yes	–
21	4.4	T2–3	Fever	5.1	140	102.3	Yes (*Pseudomonas aeruginosa* MDR)	Yes	–
22	205.6	L3–4	GIT infection	11	84	89	Yes (*Escherichia coli*)	Yes	15°
23	147.3	L5–S1	BP	9.8	45	6.4	No	Yes	–

*ASI* adjacent segment infection, *BP* back pain, *WBC* white blood cell count, *ESR* erythrocyte sedimentation rate, *CRP* C-reactive protein, *MRSA* methicillin-resistant *Staphylococcus aureus*, *MDR* multi-drug resistant, *GIT* gastrointestinal tract, *ASK* adjacent segment kyphosis

A diagnosis of spondylodiscitis was made on clinical, radiological and microbiological grounds, with patients fulfilling the following criteria:

1. Clinical symptoms suggestive of spondylodiscitis (back pain unrelieved by rest; radiating pain ± neurological deficits ± fever) with laboratory abnormalities: WBC, ESR and CRP levels.
2. Abnormal MRI (and other imaging modalities) features compatible with infection of the spine.
3. Isolation of the causative microorganism or typical histological pattern from percutaneous disk or epidural abscess puncture or biopsy.

ASI was defined similarly, with infection of the adjacent segment (vertebra or intervertebral body) after surgical treatment of the primarily treated segment(s).

### Treatment

All patients underwent operative treatment primarily and secondarily. The surgical approach was either posterior, anterior or combined anterior and posterior, with debridement, fusion and longer instrumentation. Autologous bone graft was harvested via a separate incision from the iliac crest.

Unless general health condition or intra-operative complications precluded it, all patients were mobilized with assistance on the first postoperative day. Postoperative treatment included a culture-based antimicrobial therapy, or a broad-spectrum antimicrobial therapy when no organism was isolated. This was given for a mean of 12 weeks and was stopped according to clinical, laboratory and radiological findings of recovery. After ASI surgery, antimicrobial therapy was continued for at least 12 weeks in all patients.

### Follow-up

Preoperative, postoperative and last follow-up (FU) neurological findings were assessed according to Frankel’s classification: [11].

1. ‘Complete’ (A). Paralysis, both motor and sensory, below the level marked.
2. ‘Sensory only’ (B). Some sensation present below the level of the lesion but motor paralysis complete below that level.
3. ‘Motor Useless’ (C). Some motor power present below the lesion but of no practical use to the patient.
4. ‘Motor Useful’ (D). Useful motor power below the level of the lesion.
5. ‘Recovery’ (E). Free of neurological symptoms.

The final functional outcome was completed by questionnaires including Odom’s criteria [12] which categorized patients’ satisfaction into four grades: excellent, good, fair and poor.

- Excellent: all preoperative symptoms relieved, abnormal findings unchanged or improved.
- Good: minimum residual of preoperative symptoms not requiring medication or limiting activity, and abnormal findings unchanged or improved.
- Fair: definite relief of some preoperative symptoms with others remaining unchanged or only slightly improved.
- Poor: symptoms and signs unchanged from preoperative status or worse.

### Statistical analysis

Descriptive statistics were used. Quantitative variables (e.g. age, laboratory values, operative data, interval between infections) were summarized by mean value and the standard deviation if appropriate. Qualitative demographic variables (e.g. gender and disease characteristics as well as potential prognostic factors) were summarized by counts and percentages. Analytical statistics were used to compare the preoperative and postoperative values as regards the laboratory findings. Because of the small number of cases, non-parametric tests were used, in this case the Wilcoxon signed-rank test. The same test was used to analyse the difference between the lordosis angle of the adjacent segment after primary surgery and at the time of presentation with ASI. To analyse the possible correlation between different variables and the outcomes according to Odom’s criteria, the Kruskal–Wallis test was used. Statistical significance was defined as *p* < 0.05. The statistical analysis was performed using SPSS version 13.0 (SPSS Inc., Chicago, IL, USA).

## Results

### Demography

Between 1994 and 2012, 1187 patients were surgically treated in Zentralklinik Bad Berka because of SD. Out of these, 23 (10 males, 13 females) returned with ASI (1.94 %), with a mean age 65.1 ± 10.9 years. The primary infection was lumbar in 13 (56.5 %), thoracolumbar in four (17.4 %), thoracic in three (13 %), cervical in one (4.3 %) and combined thoracic and lumbar in two cases (8.7 %). Single-level infection was found in 16 patients (65.6 %), double-level in four (17.6 %) and three levels in three (13 %). Comorbidities were found in 19 patients (82.6 %); most commonly hypertension (HT) (12 patients, 52.2 %), diabetes mellitus (DM) (7, 30.4 %), osteoporosis (5, 21.7 %) and ischemic heart disease (IHD) (5, 21.7 %) (Table 1). The general condition of the patients before the primary surgery was ASA 1 in two patients (8.7 %), ASA 2 in six (26.1 %), ASA 3 in eight (34.8 %) and ASA 4 in seven (30.4 %). This distribution reflects the generally bad condition of these patients (Table 1).

### Clinical presentation

At the time of primary infection, the main symptoms were back pain in 16 patients (69.6 %) and neurological deterioration in six (26.1 %). The average period of conservative treatment was 2.17 months before surgery (Table 2).

At the time of treatment of ASI, patients presented most commonly with recurrence of severe back pain (15 cases, 65.2 %). The mean interval between the operation of primary infection and the operation of ASI was 36.88 months.

Neurologically, one patient had Frankel grade B paraplegia (4.3 %), six patients had paraparesis grade C (26.1 %), one had grade D (4.3 %), and 15 patients (65.2 %) were neurologically free (grade E).

### Laboratory findings

The mean preoperative laboratory values were WBC 9830 ± 4743/mm^3^, ESR 77.8 ± 36.7 mm/h and CRP 94.8 ± 77.2 mg/dL. The difference in relation to the immediate postoperative values was not statistically significant (*p* = 0.813, 0.465 and 0.594, respectively). At readmission with ASI, the mean values were WBC 10,496 ± 6697/mm^3^, ESR 79.8 ± 37 mm/h and CRP 94 ± 83 mg/dL. Postoperative values did not differ significantly after ASI operation (*p* = 0.859, 0.345 and 0.889, respectively).

### Diagnostic imaging

The most common primarily involved levels were L3–4 (seven, 30.4 %), L4–5 (seven, 30.4 %) and L2–3 (five, 21.7 %). ASI most commonly involved L3–4 (seven, 30.4 %), T12–L1 (five, 21.7 %) and L2–3 (four, 17.4 %). ASI involved cranial segment in ten patients (43.5 %), caudal segment in ten (43.5 %), floating segment in two (8.7 %) and adjacent segments cranially and caudally in one case (4.3 %), mono-segmental affection in 19 cases (82.6 %), bi-segmental in seven cases (30.4 %) and multi-segmental in one case (4.3 %).

Multifocal non-contiguous spinal infection was diagnosed in four patients (17.4 %); two cervical and two thoracic spinal infections coincided with lumbar infection. An epidural abscess was found in four patients (17.4 %) and psoas abscess in seven (30.4 %).

### Primary surgery

The mean operative time was 217 ± 69.5 min with a mean blood loss of 1223 ± 710 ml. Mono- and bi-segmental spinal fusions were done in seven (30.4 %) and eight (34.8 %) patients, respectively. Three-, four- and five-segment fusions were performed in five patients (21.7 %), two (8.7 %) and one (4.3 %), respectively. Interbody fusion was done in 16 patients (69.6 %), while corpectomy was done in seven patients (30.4 %). Eight patients had bone graft only (34.8 %), and 15 had bone graft and cage (65.2 %). Minimally invasive techniques (e.g. video-assisted thoracoscopic surgery and percutaneous instrumentation) were used in six patients (26.1 %), while an open technique was used in 17 patients (73.9 %) (Table 3).

**Table 3:** Operative data of spondylodiscitis and adjacent segment infection surgical treatment

	Primary surgery	Surgery for ASI
Operative time (min)	217 ± 69.5	215 ± 106.8
Blood loss (ml)	1223 ± 710	1241 ± 587.1
Mono-segmental operation	7 (30.4 %)	0
Bi-segmental	8 (34.8 %)	6 (26.1 %)
Three segments	5 (21.7 %)	4 (17.4 %)
Four segments	2 (8.7 %)	3 (13 %)
Five segments	1 (4.3 %)	5 (21.7 %)
>Five segments	0	5 (21.7 %)
One setting operation	22 (95.7 %)	20 (87 %)
Two settings	1 (4.3 %)	3 (13 %)
Posterior approach only	1 (4.3 %)	5 (21.7 %)
Anterior approach only	1 (cervical) (4.3 %)	0
Anterior and posterior approaches	21 (91.3 %)	18 (78.3 %)
Bone graft only	8 (34.8 %)	13 (56.5 %)
Bone graft and cage	15 (65.2 %)	10 (43.5 %)
Interbody fusion	16 (69.6 %)	21 (91.3 %)
Corpectomy and fusion	7 (30.4 %)	2 (8.7 %)
Open technique	17 (73.9 %)	15 (65.2 %)
Minimally invasive technique	6 (26.1 %)	8 (34.8 %)
Microorganism		
*Staphylococcus aureus*	5 (21.7 %)	4 (17.4 %)
*Staphylococcus epidermidis*	3 (13 %)	3 (13 %)
*Pseudomonas aeruginosa*	4 (17.4 %)	3 (13 %)
*Enterococcus faecalis*	2 (8.7 %)	0
*Escherechia coli*	1 (4.3 %)	1 (4.3 %)
No organism	8 (34.8 %)	12 (52.2 %)

### Postoperative treatment

A broad-spectrum antimicrobial was started on the same day after surgery (or continued), and was shifted according to culture and sensitivity tests. The most common causative organism identified (in primary SD) was *Staphylococcus aureus*, in five patients (21.7 %). In 8 patients, no organism could be isolated (34.8 %) (Table 3).

### Surgery for adjacent segment infection

The mean operative time was 215 ± 106.8 min with a mean blood loss of 1241 ± 587.1 ml. Bi-segmental spinal fusions were done in six patients (26.1 %), while the majority of patients had long-segment fusions; five segments in five patients, and more than five segments in five patients (21.7 %). Interbody fusion was done in 21 patients (91.3 %), while corpectomy was done in only two patients (8.7 %). Ten patients had bone graft and cages (43.5 %), and 13 had bone graft only (56.5 %). Minimally invasive techniques were used in eight patients (34.8 %) and open technique in 15 patients (65.2 %) (Table 3). Of 11 patients with positive microbiological findings, eight (72.7 %) had a recurrence of the same micro-organism with multiple antimicrobial drug resistance and three (27.3 %) had a superadded infection with another organism.

### Radiological results

Marked increase in adjacent segment kyphosis (>10°) occurred in six patients (26.1 %) and screw loosening was identified in 13 patients (56.5 %) at the time of presentation with ASI (Table 2).

### Functional outcome

At the later presentation with ASI, two patients had deteriorated to grade C (8.7 %) and three had weakness grade D (13 %). The mean FU period was 69 ± 55.13 months after primary surgery. At last FU, six patients (26.1 %) were mobilized in a wheelchair with a varying degree of paraplegia (three had pre-existing paralysis). The others did not have neurological changes during the FU period (Table 1). Three patients died within 2 months after ASI operation because of sepsis and/or multi-organ failure (13 %). Subjectively, out of 18 surviving patients at the time of this study, an excellent outcome was achieved in five (27.8 %), good in eight (44.4 %), fair in four (22.2 %) and poor in one patient (5.6 %). This outcome was not significantly related to age, sex, region affected, neurological status or other variables, as statistically analysed in Table 4.

**Table 4:** Statistical analysis of different factors as regards the final functional outcome according to Odom’s criteria

Outcome (Odom’s criteria)	Excellent	Good	Fair	Poor	Died	Total	*p* value
Sex							
Male	1	3	3	1	2	10 (43 %)	0.406
Female	4	5	1	0	3	13 (57 %)	
Involvement of segments							
Monosegmental	5	5	4	1	2	17 (74 %)	0.171
Bisegmental	0	2	0	0	3	5 (22 %)	
Multisegmental	0	1	0	0	0	1 (4 %)	
General condition (ASA score)							
ASA 1 (best)	2	0	0	0	0	2 (9 %)	0.133
ASA 2	1	1	3	0	1	6 (26 %)	
ASA 3	1	4	1	0	2	8 (35 %)	
ASA 4 (worst)	1	3	0	1	2	7 (30 %)	
Preoperative neurology (Frankel grade)							
B (paralysis)	0	0	0	0	1	1 (4 %)	0.66
C	1	3	1	1	0	6 (26 %)	
D	0	0	0	0	1	1 (4 %)	
E (normal)	4	5	3	0	3	15 (65 %)	
Region of primary infection							
Cervical	0	1	0	0	0	1 (4 %)	0.793
Thoracic	1	1	0	0	1	3 (13 %)	
Thoracolumbar	1	1	1	1	0	4 (17 %)	
Lumbar	3	5	3	0	4	15 (65 %)	
Other factors							
Septicemia	1	3	0	0	4	8 (35 %)	0.119
Screw loosening	2	3	3	1	4	13 (57 %)	0.406
ASK (>10°)	0	1	1	1	3	6 (26 %)	0.092

None of these factors significantly affected the outcome of the disease

*ASA* American Society of Anesthesiologists, *ASK* adjacent segment kyphosis

## Discussion

To the best of the authors’ knowledge, no previously published study has described the prevalence of ASI after surgical fusion in SD. We conducted a PubMed/Medline search and review of the available literature up to December 2014. This phenomenon has been described only in three case reports: one lumbar in Germany [13] and two cervical in India [9, 14]. This study presents the first case series of ASI in the literature. The main limitations of this study are the variable treatment options, the wide variation in FU period and the retrospective design of the study with a low level of evidence.

Because of long conservative treatment of SD, no causative organism could be isolated in many patients previously confirmed to have this disease. The surgical approach with radical debridement, posterior stabilization and reconstruction of anterior column using expandable titanium cages is a widespread and accepted method [13]. Korovessis et al. have even shown that the use of titanium mesh cages may have a beneficial influence on eradication of infection and fusion [15]. In addition, Ruf et al. did not find any association between titanium cages and persistence or recurrence of infection [16]. The goals of surgical intervention are to preserve neurological function and to facilitate stable bony fusion without severe kyphosis. Procedures range from decompression, debridement and drainage to interbody fusion and grafting, and are decided on a case-by-case basis [2].

Based on the current study, we suggest early surgical intervention because of the higher incidence of multi-drug-resistant micro-organisms and before extension of bone destruction and expected deterioration of general condition and neurological functions of the patient. The usual surgical treatment consists of anterior debridement, reconstruction and fusion combined with open or percutaneous posterior instrumentation. This allows adequate eradication of the septic focus, resistance-adjusted antimicrobial therapy and early mobilization. Postoperative antimicrobial therapy should be immediately started (or continued) for a further 12 weeks, depending on the causative organism and culture and sensitivity examinations.

From the authors’ research into an explanation of this phenomenon, the following hypotheses are presented:

1. Haematogenous infection route: prolonged preoperative and postoperative antimicrobial treatment should have minimized the risk of re-infection via this route [13]. In this study, eight patients (34.8 %) had positive blood cultures within the FU time.
2. Direct infection of adjacent segment by intra-operatively contaminated screws. This was suspected by Lange et al., using cannulated screws [13]. No cannulated screws were used in our series, which opposes the hypothesis that bacteria are being shielded from antibiotic treatment within the cannulation of screws. We still suggest that direct contamination during surgery by faulty drilling or by cranially located screws may have a role in ASI, as also suggested by Kulkarni and Hee [14]. Seven patients had ASI with the same infecting organism as primary SD; four of these organisms had acquired more antimicrobial resistance.
3. Screw loosening is a very important finding in 13 patients (56.5 %) in this series. We assume that slowly progressing loosening of screws causes repeated micro-fractures in pedicles and endplates. These micro-fractures lead to small haematomas in bone tissue in pedicles, endplates and most importantly in the endplate–disc attachment. Subsequent infection of these haematomas in previously infected and operated spinal region is likely to occur, especially in multi-morbid patients with poor general condition.
4. Proximal junctional kyphosis (PJK) is a well-known complication of spinal instrumentation, especially with long-segment fusions [17, 18]. We assume that the adjacent segment infection is a direct cause of many cases of PJK. In the current study, six patients had marked increase in kyphosis prior to ASI. From the authors’ point of view, in cases of junctional kyphosis, ASI should be suspected and intra-operative biopsies should be sent to histopathology and microbiology for exclusion of low-grade or subclinical infection.
